# Pre-analytic decrease of phenylalanine in plasma of patients with phenylketonuria treated with pegvaliase

**DOI:** 10.1016/j.ymgmr.2024.101110

**Published:** 2024-06-21

**Authors:** Coleman Turgeon, Kari Casas, Ryan Flanagan, Amy White, Dawn Peck, Gisele Bentz Pino, April Studinski Jones, Dimitar Gavrilov, Devin Oglesbee, Matthew J. Schultz, Silvia Tortorelli, Dietrich Matern, Patricia L. Hall

**Affiliations:** a Department of Laboratory Medicine and Pathology, Mayo Clinic, Rochester, MN, USA; b Sanford Health, Fargo, ND, USA

**Keywords:** Phenylketonuria, Phenylalanine, Pegvaliase, Pre-analytical error

## Abstract

Treatment of phenylketonuria (PKU) has evolved since the initial introduction of a phenylalanine (Phe) restricted diet. The most recent option for adults affected with PKU is treatment with an alternate enzyme, phenylalanine ammonia lyase (PAL), that metabolizes excess Phe. Proper management of all patients with PKU relies on accurate measurement of Phe levels in blood, to comply with guidance intended to minimize the neurological symptoms. Recently, our laboratory was notified of discrepant results for a patient with PKU who is treated with pegvaliase. Two specimens were collected at the same time but yielded unexpectedly different Phe concentrations. After exclusion of specimen mix-ups or analytical errors, we suspected that there was residual pegvaliase activity in the specimens continuing to degrade Phe after collection. To investigate this possibility, we performed spiking studies that showed the degradation of Phe over time at ambient temperatures. Sample preparation by protein crash appears to deactivate pegvaliase and prevents further Phe degradation. However, because pegvaliase deactivation would be required immediately following blood collection, appropriate mitigation measures must be implemented, including stringent pre-analytical requirements, alternate sample matrices such as dried blood spots, or point of care testing. Until then, health care professionals need to be cautious in their interpretation of Phe levels in their patients with PKU that are treated with pegvaliase.

## Highlights

• Pegvaliase has continued action after plasma samples are collected.
• Patients with PKU taking pegvaliase may have falsely decreased phenylalanine levels.
• Phenylalanine degradation post collection is variable and unpredictable.

## 1 Introduction

Phenylketonuria (PKU, OMIM #261600) is an autosomal recessive deficiency of phenylalanine hydroxylase (PAH), an enzyme catalyzing the conversion of phenylalanine (Phe) to tyrosine (Tyr). In the absence of functional PAH, Phe accumulates in blood and tissues and causes irreversible neurological damage. PKU is a treatable disorder that can be readily identified by newborn screening. Early identification and implementation of a Phe-restricted diet can avoid the most severe symptoms associated with untreated PKU [1]. While early treatment for PKU relied entirely on dietary restriction, over the last two decades improvements have been achieved, including medications to enhance residual PAH activity [2,3], and enzyme-based treatments that metabolize Phe via alternate pathways [4,5]. Pegvaliase, is a pegylated form of phenylalanine ammonia lyase (PAL), an enzyme which converts Phe to ammonia and trans-cinnamic acid. It is the first enzyme therapy for a metabolic disorder that can also be treated by dietary modification. Pegvaliase was approved for use in 2018 for the treatment of individuals 16 years of age and older with PKU who consistently have elevated blood Phe concentrations >600 μmol/L. [5,6]

Management guidelines for PKU have evolved as knowledge has increased but have consistently focused on maintaining blood Phe levels at a level which will not cause neurological damage, typically 120 to 360 μmol/L in childhood and 120 to 600 μmol/L after the age of 12 years old. The lower cutoff of 360 μmol/L is also advised leading up to and during pregnancy [7]. Dietary management for PKU is highly individualized, and patients with PKU undergo frequent testing to monitor their blood Phe levels to ensure they are in the appropriate therapeutic range. Patients are monitored using either plasma or dried blood spots (DBS) [[8], [9], [10]]. These analyses are not typically performed in hospital laboratories, thus specimens are transported from the collection site to the performing laboratory. Much attention is focused on the upper limit of Phe concentrations to prevent irreversible neurological symptoms, however Phe is an essential amino acid and required for proper growth and development. Accordingly, overrestriction of Phe can have negative side effects, including failure to thrive, hair loss, and skin lesions [11,12]. Here, we report on the investigation of discrepant blood Phe concentrations in patients treated with pegvaliase that identified a pre-analytical decrease of Phe due to the continued action of pegvaliase in specimens after collection.

## 2 Materials and methods

Our laboratory provides monitoring for patients with PKU by comprehensive amino acid profiles, as well as measurement of only Phe and Tyr in plasma and DBS. We were contacted by a physician recently to discuss discrepant results between two plasma samples collected from a patient treated with pegvaliase. The specimens were collected at the same time, but processed into two separate plasma tubes, one for a comprehensive amino acid profile, and one for targeted measurement of Phe and Tyr. This patient receives Phe and Tyr measurements monthly, and a comprehensive amino acid profile annually. Both assays are performed using isotope labeled internal standards and liquid chromatography – tandem mass spectrometry (LC-MS/MS). The results of the initial analyses are shown in Table 1. After we were notified of the concern, we undertook an investigation to rule out common causes of discrepant results: labels on all tubes were verified, and the specimens were re-analyzed by both methods. Results of the reanalysis are also shown in Table 1. Stability studies performed during assay validation did not show significant loss of Phe during freeze thaw cycles. At this point, we suspected either contamination by or continued action of pegvaliase after collection. Communication with the patient's physician confirmed that the patient was being treated with pegvaliase.

**Table 1:** Patient Phe results that raised suspicion due to discrepant results.

	Tube Submitted for Targeted Testing		Tube Submitted for Comprehensive Analysis	
	Targeted	Comprehensive	Targeted	Comprehensive
Initial Analysis	2	NP⁎	NP⁎	143
Repeat Analysis	11	2	87	38

⁎ NP: not performed; all units μmol/L.

To investigate the hypothesis of continued pegvaliase activity ex vivo, we identified plasma samples from individuals with PKU who were confirmed to be treated with pegvaliase, suspected to be treated with pegvaliase and those who were not treated with pegvaliase (children under 16 and adults on alternate treatments). Initial Phe and Tyr results were available for all patients. For adults, we focused on patients who had extremely low levels of measured Phe concentrations. These specimens were then spiked with 250 μM of aqueous Phe, and analyzed after storage at ambient temperatures at 4 time points (0, 1, 4 and 6) hours. In addition an aliquot of each spiked plasma sample was frozen at −80 °C, and thawed for analysis at the 6 h time point. Quantitative analysis of amino acids was performed by LC-MS/MS. Plasma was combined with isotopically labeled internal standard. Following protein precipitation, the supernatant was diluted and subjected to hydrophilic interaction liquid chromatography (HILIC) for the separation of isomers with MS/MS detection of the underivatized amino acids. To determine that Phe concentrations following preparation for analysis were stabilized in samples from patients with and without pegvaliase, samples were analyzed immediately and 24 h following preparation. Our routine quality control specimens were analyzed as well. The low QC was analyzed with no alterations, and the high QC sample was spiked with 250 μM Phe to verify that there was no degradation of Phe occurring in the spiking solution over this time period. Details about the specimens included in the study are shown in Table 2. Treatment status for all adult patients was confirmed via communication with providers. As pegvaliase is not approved for use in individuals <16 years of age, treatment status was assumed for patients 2 and 3.

**Table 2:** Description of specimens included in Phe spiking study. Specimens with the same specimen ID are from the same patient.

Study ID	Description	Pegvaliase Treatment?	Spiked with 250 μM Phe?	Age	Sex	Original Phe (μM)	Original Tyr (μM)
1	Low QC	NA	No	NA	NA	NA	NA
2	PKU - Infant	No	No	6 M	Male	226	36
3	PKU - Child	No	No	8 Y	Male	283	48
4	PKU - Adult	No	Yes	18 Y	Female	5	108
5A	PKU - Adult	Yes	Yes	41 Y	Female	2	47
5B	PKU - Adult	Yes	Yes	41 Y	Female	143	42
6	PKU - Adult	Yes	Yes	27 Y	Female	1	34
7A	PKU - Adult	Yes	Yes	39 Y	Female	0	23
7B	PKU - Adult	Yes	Yes	39 Y	Female	0	30
7C	PKU - Adult	Yes	Yes	39 Y	Female	3	44
8	PKU - Adult	No	Yes	62 Y	Female	7	169
9	PKU - Adult	Yes	Yes	33 Y	Female	1	53
10	High QC	NA	Yes	NA	NA	NA	NA

## 3 Results

**Fig. 1:**
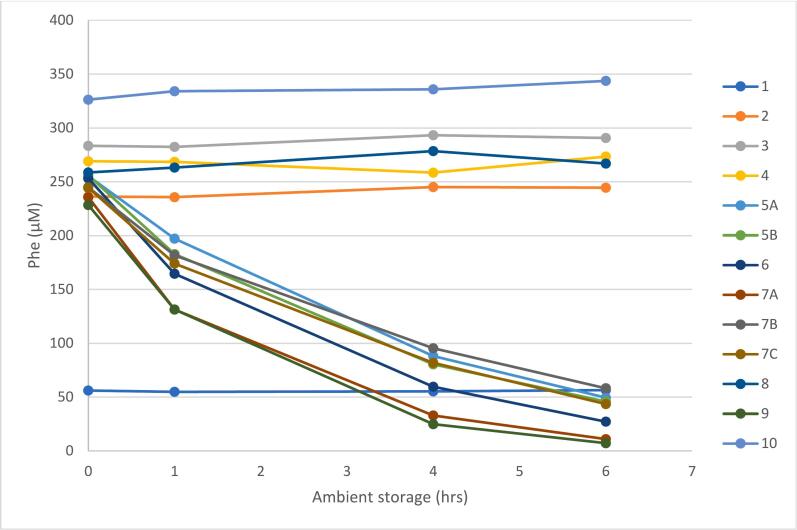
Phe concentration decreases over time in plasma specimens from patients treated with pegvaliase. Specimens 5 A, 5B, 6, 7 A, 7B, 7C and 9 are collected from treated patients and show a marked decrease in Phe over time at ambient temperatures. Specimen IDs are the same as shown in Table 2.

LC-MS/MS analysis showed steady decreases in Phe concentrations in specimens from patients confirmed to be treated with pegvaliase over the 6-h period of the study. Phe concentrations at ambient temperature over time are shown in Fig. 1. Specimens which were from untreated patients and control samples did not show a decrease in Phe concentrations over the same time period, beyond what was expected for normal variation in the analytical performance of the method (<10%). Tyr concentrations were stable for all patients at all time points, within expected method variation parameters (<10%). This was expected, as PAL does not break down Phe into Tyr. All samples from patients not treated with pegvaliase showed stable Phe concentrations, for both endogenous and spiked specimens. Initially, we selected adult patients with Phe < 10 μmol/L, and included them in our study as suspected on receiving pegvaliase treatment. Our spiking study suggested that two patients (#4 and #8) did not have residual pegvaliase activity in their plasma specimens and follow up with their providers confirmed that they were treated conventionally and without pegvaliase.

Preliminary studies were performed to identify measures which may arrest the activity of pegvaliase in an already collected plasma specimen. While we do not have a large enough dataset to explore any effect related to pegvaliase dose or timing, most recommendations are to collect specimens immediately prior to a dose. However compliance is unknown, and it is unclear if this would be enough time to clear pegvaliase activity in blood. Pegvaliase, as an unadministered medication, is stable for at least 30 days at room temperature [13]. Freezing at −80 °C arrests the activity, however we still found an average decrease of 23% between the measurement at *t* = 0 h and the specimen stored at −80 °C analyzed at *t* = 6 h for patients treated with pegvaliase. This decrease most likely represents activity of pegvaliase during the freeze/thaw process and sample preparation steps. Specimens collected from patients who were not treated with pegvaliase showed an average difference of only 1% over 6 h (Table 3). Preparation of the sample by protein crash appears to stabilize the Phe concentration, as evidenced by the re-analysis of the *t* = 0 prepared plate at *t* = 24 h (Table 3). These studies show that some mitigation of Phe loss is possible, with proper collection and storage, but there is still a significant drop in the time needed for the minimal necessary amount of sample preparation. Protein crash at the moment of collection is likely not practical, due to the number of sites at which collections take place, and the additional reagent requirements.

**Table 3:** Additional studies showing decrease in Phe concentrations when stored at −80 °C and stability after protein crash for sample preparation.

Study ID	Pegvaliase Treatment?	Spiked with 250 μM Phe?	t = 0 h Phe (μM)	t = 6 h —80C storage Phe (μM)	Difference from *t* = 0	t = 0 h prepared sample reinjected next morning Phe (μM)	Difference from initial injection
1	No	No	56	NP	NP	54	−3%
2	No	No	236	NP	NP	238	1%
3	No	No	283	NP	NP	285	0%
4	No	Yes	269	271	1%	271	1%
5A	Yes	Yes	255	209	−18%	258	1%
5B	Yes	Yes	256	208	−19%	253	−1%
6	Yes	Yes	253	189	−25%	253	0%
7A	Yes	Yes	236	159	−32%	244	4%
7B	Yes	Yes	245	208	−15%	250	2%
7C	Yes	Yes	244	195	−20%	242	−1%
8	No	Yes	259	261	1%	262	1%
9	Yes	Yes	228	160	−30%	235	3%
10	No	Yes	326	341	5%	326	0%

NP: not performed.

## 4 Discussion

Management of patients with PKU has always centered around maintaining blood Phe levels at appropriate levels to prevent neurological damage. Medications, such as pegvaliase, designed to reduce blood Phe are a recent addition to treatment options and are particularly beneficial to adult patients who are unable to manage blood Phe levels by other means. This report identifies a challenge to obtaining accurate Phe measurements in plasma of patients treated with pegvaliase. While extended storage at ambient temperatures is generally not recommended for plasma amino acid analysis, specimens do spend some unavoidable time at ambient temperatures between collection and processing. Significant residual pegvaliase activity remains in plasma after collection, and even freezing at −80 °C does not permanently inactivate the enzyme as we found a reduction of measured Phe concentrations of approximately 20% after thawing frozen specimens before preparation for analysis. Accordingly, routine repeat analyses, for example due to a quality control failure, should be treated with extreme caution, and it may be necessary to obtain a new sample rather than relying on the established stability metrics determined during assay validation. Point of care (POC) approaches to PKU management have been explored, and may benefit particularly patients treated with pegvaliase [14,15]. Interestingly, a POC approach utilizing PAL and ammonia measurement has been proposed, although its effectiveness in the context of pegvaliase treatment has not been established [16].

We were unable to identify a single event that caused the initial discrepant results between two samples collected at the same time. Perhaps the sample with initially lower Phe value was exposed longer to room temperature, either due to an increased time (relative to the other tube) before freezing and/or while being thawed and processed in the laboratory for testing.

Additional studies are required to evaluate mitigation strategies of ex vivo pegvaliase activity and its clinical relevance. Our literature search did not identify other reports of unexpectedly low Phe concentrations in patients treated with pegvaliase that were not assumed to reflect the patient's circulating Phe concentration at the time of collection. Indeed, while hypophenylalaninemia is a recognized possible effect of treatment, recent management guidelines advise dietary adjustment as a countermeasure, and do not mention the possibility of a falsely decreased result, beyond requiring two low measurements consecutively [17,18]. Moreover, no specific blood collection protocols are recommended that would suggest there was concern for or realization of continued action of pegvaliase in blood and plasma specimens. Reassuringly, there are no reports of improper pegvaliase dosing which can likely be attributed to the fact that treatment decisions are not based solely on Phe concentrations measured in plasma and (measured) Phe concentrations down to 31 μmol/L are considered acceptable [19]. Similarly, our study was not prompted by a concern for overtreatment resulting in hypophenylalaninenia but for our laboratory's ability to measure reliable Phe concentrations in aliquots of blood from the same collection. Potential mitigation strategies are varied and will require extensive testing and experimentation. Some options are: exploiting and enhancing the stabilizing features of filter paper, chemical inhibitors of pegvaliase activity, or alternate analyses to include downstream metabolites such as trans-cinnamic acid to calculate a value that correlates with the blood Phe levels.

In conclusion, especially unexpectedly low Phe concentrations in a patient treated with pegvaliase should be interpreted with caution, and not assumed to reflect circulating Phe. Adjustment of diet or other treatments based on extremely low Phe values may result in higher than intended blood Phe concentrations. Conversely, measured Phe concentrations at the upper limit of the target range should not be assumed to reflect sufficiently appropriate treatment. Moreover, the continued ex-vivo activity demonstrated by pegvaliase administered to these patients should be a consideration for other situations where amino acid metabolism may be altered by exogenous enzymes, such as emerging cancer therapies [20]. If monitoring the targeted metabolite is a key factor in the treatment regimen, steps should be taken to ensure that this can be done reliably, including the consideration to alternative specimen types such as blood dried on filter paper. This preliminary study is a reminder of the importance of pre-analytical variables in the accuracy of laboratory measurements, especially in the development of new therapies where associated biomarkers of disease are used to monitor treatment.

## Funding

This research did not receive any specific grant from funding agencies in the public, commercial, or not-for-profit sectors.

## CRediT authorship contribution statement

**Coleman Turgeon:** Writing – review & editing, Methodology, Investigation, Formal analysis, Conceptualization. **Kari Casas:** Writing – review & editing, Investigation, Conceptualization. **Ryan Flanagan:** Writing – review & editing, Investigation. **Amy White:** Writing – review & editing, Investigation. **Dawn Peck:** Writing – review & editing, Investigation. **Gisele Bentz Pino:** Writing – review & editing, Investigation. **April Studinski Jones:** Writing – review & editing, Investigation. **Dimitar Gavrilov:** Writing – review & editing, Investigation. **Devin Oglesbee:** Writing – review & editing, Investigation. **Matthew J. Schultz:** Writing – review & editing, Investigation. **Silvia Tortorelli:** Writing – review & editing, Investigation. **Dietrich Matern:** Writing – review & editing, Writing – original draft, Methodology, Investigation, Conceptualization. **Patricia L. Hall:** Writing – review & editing, Writing – original draft, Methodology, Investigation, Formal analysis, Data curation, Conceptualization.

## Declaration of competing interest

The authors declare no conflict of interest related to this work.

## Data Availability

All data used are in the article
